# A new Chinese *Pseudoogeton* species and key to the species of the genus (Coleoptera, Tenebrionidae, Amarygmini)

**DOI:** 10.3897/zookeys.553.6654

**Published:** 2016-01-14

**Authors:** Sai-Hong Dong, Guo-Dong Ren

**Affiliations:** 1 The Key Laboratory of Zoological Systematics and Application, College of Life Sciences, Hebei University, Baoding 071002, P. R. China

**Keywords:** Tenebrionidae, Pseudoogeton, new species, China, key to species

## Abstract

*Pseudoogeton maoxianum* **sp. n.** is described from Sichuan, China. A key to the males of the species of *Pseudoogeton* Masumoto, 1989 is presented.

## Introduction

*Pseudoogeton* (Tenebrionidae: Amarygmini) was established by Masumoto (1989) with *Pseudoogeton amplipennis* (Fairmaire, 1897) as the type species. The genus is similar to *Plesiophthalmus* Motschulsky, 1858, but it can be distinguished from the latter by the absence of hind wings or shortened hind wings.

Although Bremer and Lillig (2014) are of the opinion that the absence of hind wings is not a satisfactory character state to separate the genus from *Plesiophthalmus*; still, *Pseudoogeton* is retained as a separate genus until other characters are found to justify its status or to merge it with *Plesiophthalmus*.

At present the genus contains six valid species described by Fairmaire (1891, 1897) and Masumoto (1981, 1989, 1996, 2010). They are distributed in the mountains of China (*Pseudoogeton amplipenne* (Fairmaire, 1897), *Pseudoogeton gebieni* Masumoto, 1989, *Pseudoogeton ovipenne* (Fairmaire, 1891) and *Pseudoogeton uenoi* (Masumoto, 1981)), the Ryukyu Island (*Pseudoogeton kimurai* Masumoto, 1996) and Laos (*Pseudoogeton endoi* Masumoto, 2010).

Working on Chinese specimens of the genus deposited in the Museum of Hebei University (MHBU), Baoding, a new species was found, which was collected by the authors in Sichuan Province of China in 1999. In this paper the new species is described and a key to males of the species of *Pseudoogeton* is presented.

## Material and methods

The photos were taken with a Leica DFC 450 digital microscope camera attached to Leica M205A stereomicroscope. The aedeagus was dissected, cleared in 5% NaOH solution, and placed in glycerin for observation and imaging. Images were edited using Adobe Photoshop CS6. The terminology follows Masumoto (2010). All measurements given are in millimeters. The type specimens are deposited in MHBU, Baoding, China.

## Results

### Key to the species of *Pseudoogeton* (males)

*Pseudoogeton ovipenne* from Hubei whose holotype is female is not in the key.

**Table:** 

1	Hind wings shortened	**2**
–	Hind wings absent	**3**
2	Pronotum approx. as wide as long; apicale of aedeagus arrowheaded (Figs 25–26, in Masumoto 1989). China (Taiwan)	***Pseudoogeton uenoi***
–	Pronotum approx. 1.4 times as wide as long; apicale of aedeagus simply short fusiform. Japan	***Pseudoogeton kimurai***
3	Body blackish brown, with ferruginous tinge, dorsal surface strongly shining; apicale of aedeagus elongated equilateral triangular, lateral sides gradually narrowed forward. Laos	***Pseudoogeton endoi***
–	Black, or with violet and sericeous tinge; lateral sides of apicale of aedeagus abruptly narrowed forward	**4**
4	Pronotum widest in middle (Fig. 3); aedeagus stouter, apicale length/width < 1.5 (Figs 5–7). China (Sichuan)	***Pseudoogeton maoxianum* sp. n.**
–	Pronotum widest behind middle; aedeagus slender, apicale length/width > 1.5	**5**
5	Dorsal surface of body with violet tinge; pronotum hemispherical; striae on elytra fine and shallow, intervals almost not punctate. China (Sichuan)	***Pseudoogeton gebieni***
–	Dorsal surface of body black, pronotum moderately convex and transverse; striae on elytra strong and deep, intervals with clear punctures. China (Sichuan)	***Pseudoogeton amplipenne***

**Figures 1–9.**
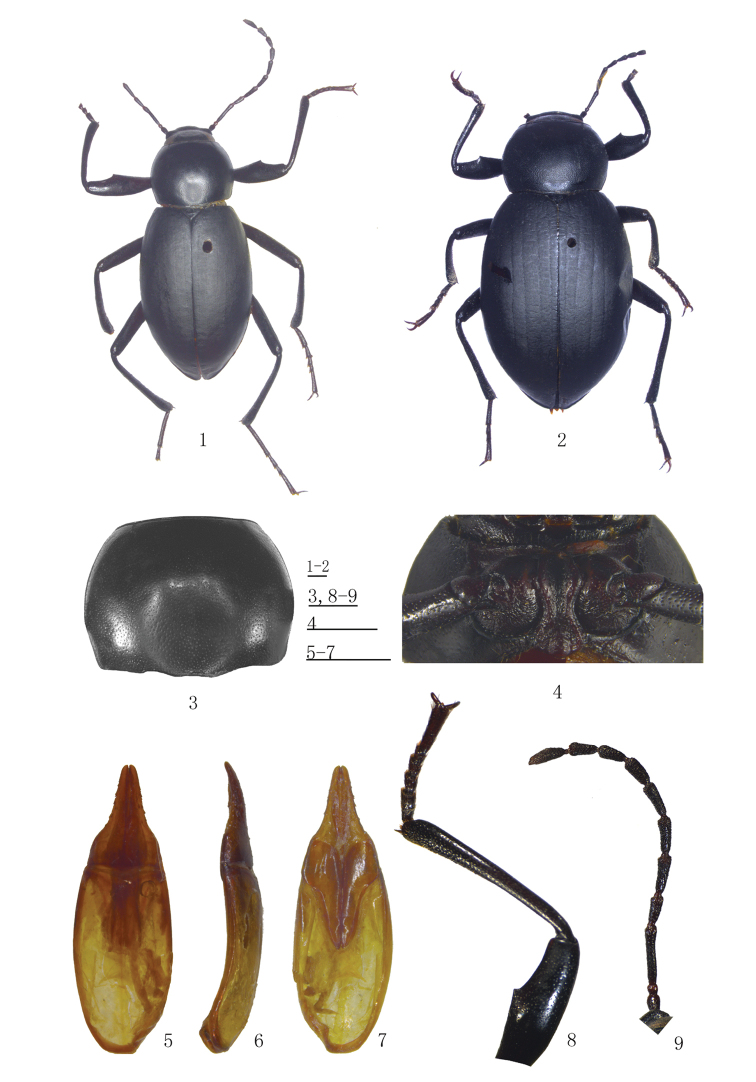
*Pseudoogeton maoxianum* sp. n. **1** Habitus, male **2** Habitus, female **3** Pronotum **4** Prosternal process **5** Aedeagus in dorsal view **6** Aedeagus in lateral view **7** Aedeagus in ventral view **8** Foreleg **9** Antenna. Scale bars: 1 mm.

### Pseudoogeton maoxianum sp. n.

Taxon classificationAnimaliaColeopteraTenebrionidae

http://zoobank.org/C3E7E346-28DE-4EBC-A8A6-62CBED3F2AAB

#### Type specimens.

Holotype ♂ (MHBU): CHINA, Sichuan, Maoxian County, Mt. Xiaomiaoshan, 1600m, 22.viii.1999, leg. Guo-Dong Ren & Sai-Hong Dong. Paratype: 1♀ (MHBU): same data as holotype [Transliterated from Chinese labels].

#### Distribution.

China (Sichuan).

#### Diagnosis.

The new species is characterized by the following: pronotum nearly hemispherical, widest in middle; elytra strongly convex, with fine strial puncture; apicale of aedeagus stouter, ratio of length/width = 1.4; basale 1.9 times longer than apicale.

#### Description.

*Male*. Wingless; body oblong oval (Fig. 1), dorsum strongly convex, black; elytra with sericeous tinge, pronotum and legs more lustrous than elytra.

Clypeus transverse, with dense punctures. Frontoclypeal suture fine and straight. Genae relatively small, roundly protruded laterad. Eyes small, reniform, distance between them approximately 2.3 times their own diameter. Mentum trapezoidal. Gula widely triangular. Terminal maxillary palpomeres securiform. Antennae filiform (Fig. 9), reaching over half of elytra; length ratio of antennomeres 1 to 11 as 0.33: 0.20: 1.24: 0.59: 0.78: 0.69: 0.69: 0.64: 0.57: 0.61: 0.85.

Pronotum (Fig. 3) convex, almost hemispherical, 1.3 times as wide as long, widest in middle, roundly narrowed anteriorly and posteriorly, anterior margin slightly arcuate, finely beaded, posterior margin noticeably produced, lateral margins finely beaded, visible in dorsal view throughout their whole length, anterior and posterior corners obtuse in lateral view; disc with tiny and sparse punctures.

Scutellum widely triangular, with a few punctures.

Elytra ovate, approximately 1.5 times as long as wide, approximately 2.6 times longer and 1.3 times wider than pronotum. Dorsum convex, maximum height at basal third, widest at basal 2/5, lateral sides roundly narrowed anteriorly and posteriorly. Disc with rows of tiny and sparse punctures, their distance equal to 3–4 times of puncture diameter; intervals flat and wide, transversely micro-aciculate, with extremely tiny punctures; lateral margins finely beaded.

Prosternum with a deep median groove between coxae. Prosternal process (Fig. 4) strongly bent upwards, with apophysis near apex.

Legs slender and rather long. Anterior edge of profemora (Fig. 8) with acute spine in apical third. Protibiae moderately curved, slightly widened and haired in apical half. Ratio of length of pro-, meso- and metatarsomeres 1 to 5 (or 4, metatarsomeres) as 0.60: 0.42: 0.42: 0.29: 1.13; 1.13: 0.63: 0.45: 0.28: 1.14; 1.93: 0.71: 0.46: 1.18. Claws sharp, falciform.

Abdominal ventrites with microscopic punctures and setae, ventrite V slightly emarginate at apex.

Aedeagus fusiform (Figs 5–7), 3.5 mm long. Apicale elongate, lateral sides serrate, ratio of length/width = 1.4. Basale 1.9 times longer than apicale.

*Female*: Body stouter than male (Fig. 2), pronotum 1.5 times as wide as long, elytra ovate, about 1.4 times as long as wide, about 3.0 times longer and 1.4 times wider than pronotum; prosternum groove between the coxae shallow.

Body length: ♂: 12.9 mm; ♀: 15.2 mm. Body width: ♂: 5.7 mm; ♀: 7.8 mm.

#### Etymology.

This specific epithet is derived from the type locality, Maoxian County, Sichuan Province, China.

## Supplementary Material

XML Treatment for Pseudoogeton maoxianum
